# Illuminating the invisible: translational biophotonics for contemporary caries detection and biological decision-making

**DOI:** 10.3389/froh.2026.1905754

**Published:** 2026-07-17

**Authors:** Hervé Tassery, Virgnie Pilliol, Elodie Terrer, Amel Slimani

**Affiliations:** 1 Institut Hospitalo-Universitaire (IHU) Méditerranée Infection, Aix-Marseille University, Marseille, France; 2 Microbes, Evolution, Phylogeny and Infections (MEPHI), Aix-Marseille University, Marseille, France; 3 Department of Restorative Dentistry and Endodontics, Ecole de Médecine Dentaire, Aix-Marseille University, Marseille, France; 4 LBN, University of Montpellier, Montpellier, France; 5 Department of Restorative Dentistry and Endodontics, Faculty of Dentistry, University of Montpellier, Montpellier, France; 6 Department of Restorative Dentistry and Endodontics, CHU of Montpellier, CSD M-F Calais, Montpellier, France

**Keywords:** biophotonics, dental caries, detection technologies, diagnostic, fluorescence imaging, minimally invasive dentistry

## Abstract

Dental caries remains the most prevalent chronic condition globally, yet conventional diagnostic methods often fail to detect early lesions. Biophotonics, the study of light–tissue interactions, provides a new diagnostic pathway by exploiting the inherent optical properties of enamel and dentin. Over the past decades, research has connected laboratory spectroscopy, molecular chemistry and clinical imaging to define how light-based diagnostics can reveal the earliest biochemical signatures of demineralization. Conventional diagnostic methods, including visual examination and radiography, remain indispensable but are limited in their ability to detect early subsurface lesions, assess lesion activity and characterize the biological status of affected tissues. Recent advances in biophotonics can be exploited to generate clinically relevant biomarkers of disease initiation and progression. This review examines the translational pathway linking fundamental photonic phenomena in dental hard tissues to contemporary caries detection technologies and biologically guided treatment strategies. Emphasis is placed on light transmission and fluorescence-based technologies, fluorescence spectroscopy, Raman spectroscopy, multiphoton microscopy, second harmonic generation (SHG), two-photon excited fluorescence (2PEF) and optical coherence tomography (OCT). Experimental investigations have demonstrated that dentinal collagen degradation is associated with a progressive reduction in the SHG/2PEF ratio, while fluorescence and Raman studies have identified porphyrin derivatives and advanced glycation end-products as major contributors to the red fluorescence observed in active carious lesions. Beyond diagnosis, contemporary developments in minimally invasive dentistry increasingly integrate photonic biomarkers into the Bioactive Dental Concept, where lesion activity, cavitation status and individual caries risk guide the selection of preventive, minimally invasive and restorative interventions. Within this framework, photonic technologies may serve not only as diagnostic adjuncts but also as biological decision-support tools. Future integration of multimodal imaging, artificial intelligence and bioactive restorative materials may further enhance the precision and personalization of caries management. This evolution exemplifies the broader potential of biophotonics to bridge fundamental optical science and clinical oral healthcare.

## 1 Introduction

Dental caries remains one of the most prevalent chronic diseases worldwide and affects individuals across all age groups despite significant advances in preventive dentistry and public health interventions (1, 2). Contemporary understanding recognizes caries as a behavioral and a dynamic biofilm-mediated disease characterized by repeated cycles of demineralization and remineralization occurring at the tooth–biofilm interface (3–6). Consequently, disease progression is no longer viewed as a linear process inevitably leading to cavitation but rather as a biologically dynamic continuum influenced by microbial, dietary, behavioral, and host-related factors (7).

These conceptual advances have profoundly altered the goals of diagnosis. Historically, caries detection focused primarily on identifying cavitated lesions requiring restorative intervention. Modern cariology instead emphasizes early detection, lesion activity assessment, risk-based management, and preservation of healthy dental tissues (8). This evolution is reflected in contemporary frameworks such as CAMBRA™ (Caries Management By Risk Assessment) Minimum Intervention Dentistry (MID), the International Caries Classification and Management System (ICCMS™), CariesCare International® and the Minimum Intervention Oral Healthcare (MIOC) model (8–11).

The success of these approaches depends heavily on the availability of diagnostic systems capable of identifying disease at its earliest stages and providing clinically meaningful information regarding lesion behavior.

The 2024 consensus recommendations published by the Organization for Caries Research (ORCA) and the European Federation of Conservative Dentistry (EFCD) emphasize that diagnosis of carious lesions relies on a combined approach, associating visual-tactile examination, radiography and complementary tools when necessary (12). Conventional diagnostic methods remain indispensable but exhibit important limitations. Visual-tactile examination is constrained by anatomical complexity and human visual acuity, while radiography provides primarily structural information and offers limited insight into lesion activity or tissue biology (13).

These limitations have stimulated the development of adjunctive technologies capable of interrogating dental tissues at structural, biochemical, and functional levels. Biophotonics has emerged as one of the most promising fields addressing this challenge. Broadly defined, biophotonics encompasses the application of optical science and photonic technologies to biological systems for diagnosis, monitoring and treatment. In dentistry, biophotonic approaches exploit interactions between light and dental tissues to generate information regarding mineralization, collagen organization, bacterial metabolism, tissue integrity and disease activity. Unlike conventional diagnostic methods, photonic technologies have the potential to reveal biological processes that remain invisible to routine clinical examination (13).

The development of dental biophotonics has been driven by a growing understanding of the intrinsic optical properties of enamel and dentin. Changes in mineral content, collagen organization, bacterial colonization and tissue degradation alter light scattering, fluorescence emission, Raman signatures and nonlinear optical responses (14–18). These changes generate measurable optical biomarkers that can be exploited for diagnostic purposes.

Protoporphyrin, porphyrins (I,IX) produced by bacterial metabolism, advanced glycation end-products (AGEs) accumulated within degraded collagen matrices, and alterations in collagen architecture measurable through second harmonic generation have all been implicated in the optical phenotype of carious tissues. Such findings have provided a mechanistic foundation for translating laboratory observations into clinical imaging technologies (15–21).

Today, a diverse range of commercial systems exploit these principles. Fluorescence-based technologies have become established adjuncts in caries diagnostics. Similarly, near-infrared transillumination (NILT), optical coherence tomography (OCT), photothermal radiometry and emerging multimodal systems continue to expand the diagnostic capabilities available to clinicians (13). Importantly, the role of these technologies is evolving. Contemporary evidence increasingly suggests that optical biomarkers may contribute not only to lesion detection but also to assessment of lesion activity, cavitation status, cleanability and progression risk. These developments support a transition from purely lesion-centered diagnosis toward biological decision-making, in which photonic information contributes to understanding disease behavior rather than simply identifying structural defects (9, 13).

This article is a narrative thematic review that synthesizes evidence from basic optical science, molecular spectroscopy and clinical dentistry to examine the translational pathway linking photonic properties of dental hard tissues to contemporary caries detection technologies and biological decision-making.

By exploring these interconnected dimensions, this review seeks to provide a comprehensive overview of the translational dental biophotonics and identify future directions for research and clinical implementation.

## 2 Methodology and scope of the review

This article is a narrative thematic review with a translational focus. Its objective is to synthesize current knowledge on the photonic properties of dental hard tissues and their translation into clinically applicable technologies for caries detection and biological decision-making. A literature search was conducted using PubMed/MEDLINE, Scopus, Web of Science, and Google Scholar. Publications from the fields of dental biophotonics, optical diagnostics, cariology and minimally invasive dentistry were considered. The search included combinations of the following keywords: biophotonics, dental caries, fluorescence imaging, Raman spectroscopy, multiphoton microscopy, second harmonic generation, two-photon excited fluorescence, optical coherence tomography, near-infrared transillumination, photonic biomarkers, lesion activity, and minimum intervention dentistry. Priority was given to peer-reviewed articles published in English, including original research papers, systematic reviews, consensus statements that contributed significantly to the understanding of light–tissue interactions and their clinical applications in cariology. Studies focusing on the molecular and structural origins of optical signals, validation of optical biomarkers and the development of clinical diagnostic technologies were preferentially included.

## 3 Photonic properties of dental hard tissues

### 3.1 Enamel optics: a highly mineralized photonic medium

The optical behavior of enamel is primarily determined by its highly mineralized composition and hierarchical microstructure. Enamel consists of approximately 96% hydroxyapatite, 3% water and less than 1% organic material, making it the most mineralized tissue in the human body. Hydroxyapatite crystallites are organized into prisms and interprismatic regions that influence light propagation through the tissue (15–20). Sound enamel exhibits relatively low absorption and weak intrinsic fluorescence because of its limited organic content. Light transmission is largely governed by scattering phenomena associated with the size, orientation, and distribution of hydroxyapatite crystals. The refractive index of healthy enamel remains relatively homogeneous, permitting efficient transmission of visible and near-infrared light (19).

During the early stages of caries development, acid-mediated dissolution of hydroxyapatite creates microscopic porosities within the enamel structure. These pores become filled with air or water, generating refractive index discontinuities that substantially increase light scattering. As scattering increases, enamel progressively loses translucency and appears clinically as a white spot lesion. This optical phenomenon forms the basis of several modern diagnostic technologies, including transillumination and quantitative fluorescence systems. The optical response of enamel is therefore closely linked to mineral content and crystal organization. Importantly, changes in scattering frequently precede cavitation, allowing photonic technologies to identify lesions at stages that may remain undetectable by conventional radiography (13). The presence of collagen and dentinal tubules significantly influences optical properties. Dentin demonstrates stronger autofluorescence, greater scattering and a broader range of nonlinear optical responses than enamel (15–17, 19).

### 3.2 Dentin optics: a mineral—organic composite tissue

In contrast to enamel, dentin exhibits considerably more complex optical behavior because of its composite composition. Dentin contains approximately 70% mineral, 20% organic matrix, and 10% water by weight. Type I collagen (non-centrosymmetric 3D conformation) constitutes the principal organic component and forms a three-dimensional scaffold surrounding hydroxyapatite crystallites (15–19). Furthermore, the anisotropic architecture of dentinal tubules introduces directional variations in light propagation and scattering. Caries progression within dentin is associated with simultaneous mineral dissolution and collagen degradation. Consequently, dentinal lesions generate optical signatures that reflect both structural and biochemical alterations. This dual sensitivity to mineral and organic changes makes dentin particularly attractive for photonic investigations (13, 16, 18, 20–22).

A major implication of dentin optics is that optical signals may provide information regarding tissue vitality and biological activity rather than merely lesion depth. Such information is highly relevant within contemporary minimally invasive and biologically driven cariology (12, 13, 23).

### 3.3 Systems based on intrinsic biological optical signals

These tools provide information regarding molecular composition, collagen organization and tissue degradation.

#### 3.3.1 *In vitro* second harmonic generation, two-photon excited fluorescence and fluorescence microscopy

One of the most important advances in dental biophotonics has been the application of multiphoton microscopy to the study of dentinal tissues (16, 17, 19, 24). Multiphoton microscopy exploits nonlinear optical interactions generated by ultrafast near-infrared laser excitation. Two complementary signals are particularly relevant to dental hard tissues:

- Second Harmonic Generation (SHG)
- Two-Photon Excited Fluorescence (2PEF)

SHG arises from highly ordered non-centrosymmetric molecular structures. Type I collagen fulfills these conditions and therefore generates strong SHG signals when intact. Consequently, SHG serves as a sensitive indicator of collagen organization and structural integrity. By contrast, 2PEF originates from endogenous fluorophores excited through simultaneous absorption of two photons. Changes in biochemical composition, collagen degradation products and tissue alteration influence fluorescence intensity (16, 17, 19, 24).

Experimental studies have demonstrated that healthy dentin exhibits strong SHG and relatively lower fluorescence, whereas progressive caries causes SHG intensity to decrease and 2PEF intensity to increase. The ratio between SHG and 2PEF therefore reflects the balance between collagen organization and biochemical degradation. Importantly, reductions in SHG/2PEF ratios have been shown to correlate with increasing lesion severity and ICDAS classifications, suggesting that this parameter may function as a quantitative biomarker of dentinal tissue degradation (16, 17).

Red fluorescence in carious tissues, revealed under fluorescence microscopy across ICDAS stages, was found to be primarily associated with Protoporphyrin IX, with possible contributions from Porphyrin I and Pentosidine. Pentosidine, an advanced glycation end-product (AGE) formed through the Maillard reaction, may contribute to the fluorescence changes observed during caries progression. These findings support the application of fluorescence-based technologies for the detection and monitoring of dental caries (18).

#### 3.3.2 *In vitro* Raman signatures and molecular characterization

Raman spectroscopy provides a complementary approach for investigating the molecular composition of dental tissues. Unlike fluorescence imaging, which provides broad optical contrast, Raman spectroscopy identifies specific vibrational signatures associated with mineral and organic components (15, 20). The phosphate *ν*1 band near 960 cm^−1^ serves as a marker of hydroxyapatite content and crystallinity, while carbonate and amide bands provide information regarding mineral substitution and collagen structure. Raman mapping studies have demonstrated significant differences between sound and carious tissues. Carious dentin was characterized by reduced phosphate intensity (*ν*1 PO_4_^3−^, ∼959 cm^−1^), increased carbonate bands (∼1,069–1,102 cm^−1^), marked enhancement of pentosidine (∼1,550 cm^−1^) and AGE-related bands (1,550–1,690 cm^−1^), together with alterations in amide III (1,243–1,275 cm^−1^), amide I (∼1,665 cm^−1^), and C–H/O–H stretching vibrations (2,948–2,998 cm^−1^), reflecting demineralization and collagen modification associated with the Maillard reaction (15, 20, 21).

Although Raman spectroscopy remains primarily a research tool because of instrumentation requirements and acquisition times, it provides critical molecular validation for many photonic biomarkers currently used in clinical practice.

#### 3.3.3 *In vitro* optical coherence tomography (OCT)

*In vitro* optical coherence tomography (OCT) has emerged as one of the most promising technologies in dental biophotonics (25). Using low-coherence interferometry, OCT generates high-resolution cross-sectional images of dental tissues without ionizing radiation. Unlike fluorescence systems that emphasize biochemical information, OCT primarily provides structural information regarding enamel integrity, lesion depth, internal morphology, restoration margins, secondary caries and crack propagation. A major advantage of OCT is its ability to detect subsurface lesions before cavitation becomes clinically apparent (26). These characteristics have led many investigators to describe OCT as an “optical biopsy” of dental tissues.

## 4 Clinical translation of photonic biomarkers into caries detection technologies

### 4.1 Beyond sensitivity and specificity: towards clinically relevant optical diagnostics

The translation of fundamental biophotonic discoveries into clinical dentistry has generated a wide range of optical technologies capable of detecting and characterizing dental caries. Historically, the diagnostic performance of these systems has been evaluated primarily through sensitivity and specificity. While these metrics remain important, they do not necessarily reflect the clinical usefulness of a technology during daily patient care (Table 1) (27).

**Table 1:** Sensitivity and specificity of the main complementary photonic caries detection devices (28, 29).

Device	Sensitivity	Specificity
QLF®	0.5–0.68	0.7–0.9
Spectra®/VistaCam®	0.92	0.37
SIROInspect®	0.94	0.83
DIAGNOdent®	0.87	0.5
Soprolife®	0.93	0.87

Contemporary cariology has evolved substantially from a lesion-detection paradigm toward a biologically driven approach emphasizing lesion activity, cavitation, cleanability, progression risk and longitudinal monitoring (8–10). Consequently, clinicians require information extending beyond the simple presence or absence of disease. Recognizing these limitations, the Bioactive Dental Concept proposed a clinically oriented framework for evaluating caries detection technologies based on criteria directly relevant to patient management rather than diagnostic accuracy alone (9). These criteria include:

- Detection of cavitation
- Assessment of lesion activity
- Evaluation of cleanability and accessibility
- Images and videos documentation
- Magnification capability in pre-per post restorations
- Longitudinal monitoring
- Operative usefulness

Within this framework, the value of photonic technologies lies not merely in lesion detection but in their ability to provide biologically meaningful information that supports clinical decision-making. From a translational perspective, optical diagnostics have progressively evolved from imaging tools into biological assessment systems capable of revealing structural, biochemical, and functional aspects of disease.

### 4.2 Enhanced visual diagnostics: magnification and optical augmentation

Although advanced optical technologies have transformed caries detection, visual examination remains the cornerstone of clinical diagnosis. However, visual inspection is constrained by the physiological limitations of human vision. At conventional working distances, the human eye is unable to reliably discriminate structures below approximately 100–150 μm, rendering subtle enamel defects and early cavitation difficult to detect (13).

#### 4.2.1 Optical magnification

Magnification systems improve visualization of morphological features associated with early caries. Dental loupes typically provide magnification ranging from 3.5× to 6× and are frequently combined with high-intensity LED illumination. Several studies have demonstrated improvements in lesion detection and diagnostic confidence when magnification is used during visual examination (30, 31). Enhanced visualization facilitates identification of the surface texture changes, loss of enamel luster, initial cavitation, marginal defects and plaque-retentive areas. These features are highly relevant within activity-based caries assessment. A digital version of loop was launched recently (ARloupe, Bewelltechs, China) given the opportunity to record images and videos with high level of magnification.

#### 4.2.2 Fluorescence-assisted magnification

**Figure 1:**
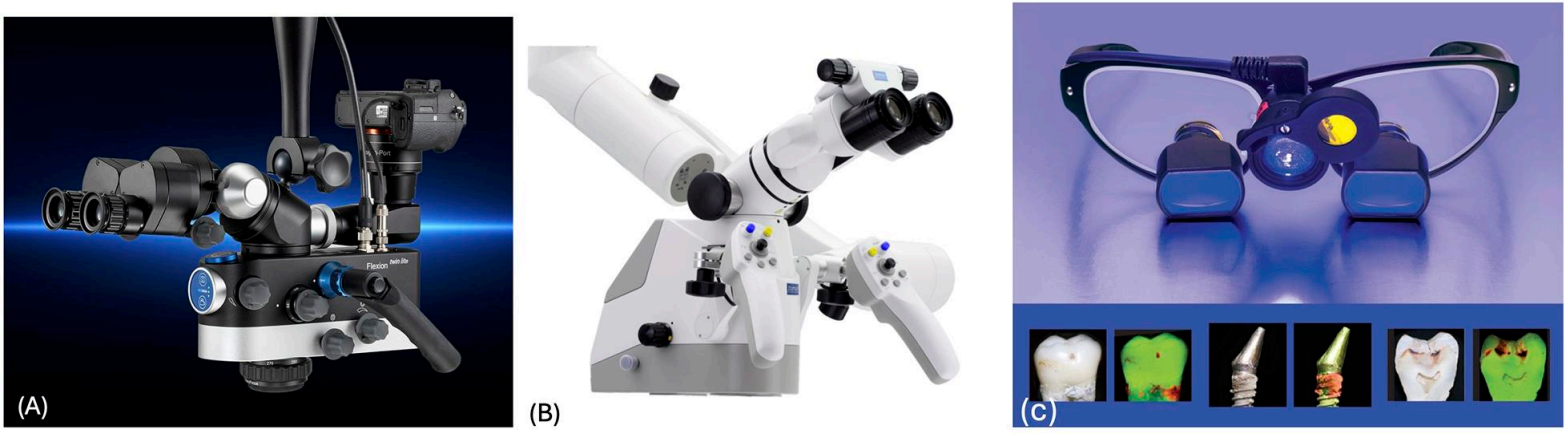
Fluorescence-assisted magnification devices. (**A**) Microscope Flexion Twin—JOptic (Germany) [Source: www.cj-optik.de]; (**B**) Microscope OMS3200 Pro -Zumax (China) [Source: www.zumaxmedical.com]; (**C**) REVEAL Loupe—Design For Vision (USA) [Source: www.designsforvision.com].

More recently, fluorescence-assisted visualization systems have emerged that combine optical magnification with fluorescence imaging notably featuring operating microscopes or magnifying loupes equipped with fluorescence excitation (Figure 1).

Fluorescence-assisted magnification combines high-resolution optical magnification with fluorescence imaging to enhance the visualization of early carious lesions and subtle changes in dental hard tissues. These systems typically use blue or violet LED illumination (approximately 405–450 nm) to excite endogenous fluorophores within enamel, dentin and bacterial metabolites. The emitted fluorescence, characterized by longer wavelengths, is separated from the excitation light by optical filters and recorded by a high-resolution digital sensor integrated into the intraoral camera or operating microscope. Sound enamel and dentin exhibit predominantly green fluorescence resulting from their intrinsic autofluorescence, whereas carious tissues produce an increased red fluorescence due to the accumulation of bacterial porphyrins and advanced glycation end-products associated with collagen degradation. The combination of magnification and fluorescence imaging enables clinicians to simultaneously assess surface morphology and biological changes that are not readily detectable under conventional white-light illumination.

Fluorescence-loupe allows the clinician to conduct operative procedures with optional fluorescence guidance from detection to caries removal to treatment completion. Furthermore, a recent clinical study illustrated the use of fluorescence loupes, Reveal® DFV, as an adjunctive tool for the chairside evaluation of soft tissues inflammation, potentially malignant and malignant oral lesions (Figure 1C) (22, 32).

**Figure 2:**
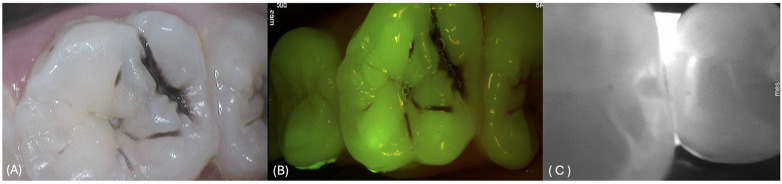
DIAGNOcam® HD (Kavo, Germany) intraoral camera. **(A)** Daylight image; **(B)** Fluorescence image; **(C)** transillumination image revealing a proximal lesion.

**Figure 3:**
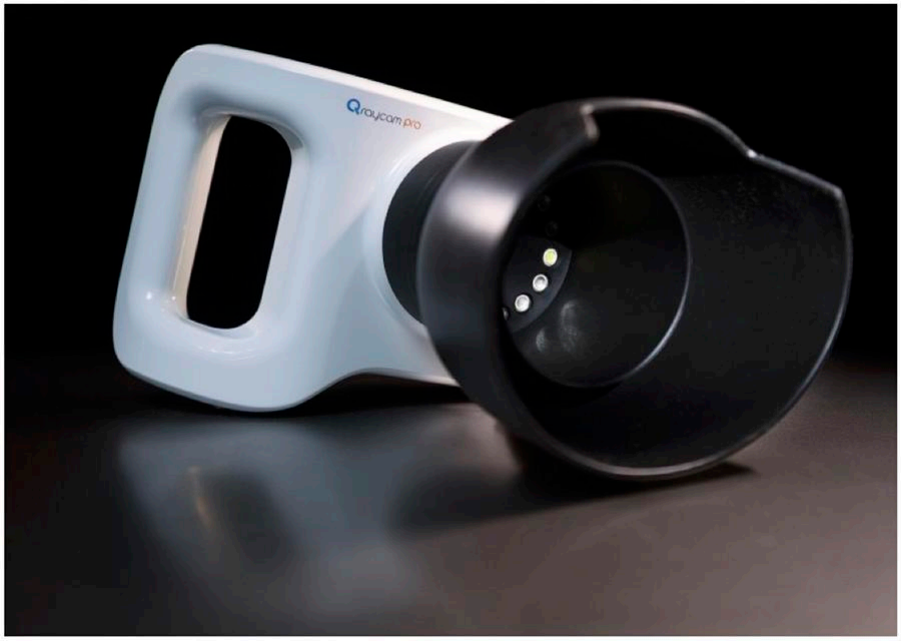
Qraycam Pro® (Korea). QLF dental caries and plaque diagnostic device [www.aiobio.co.kr].

**Figure 4:**
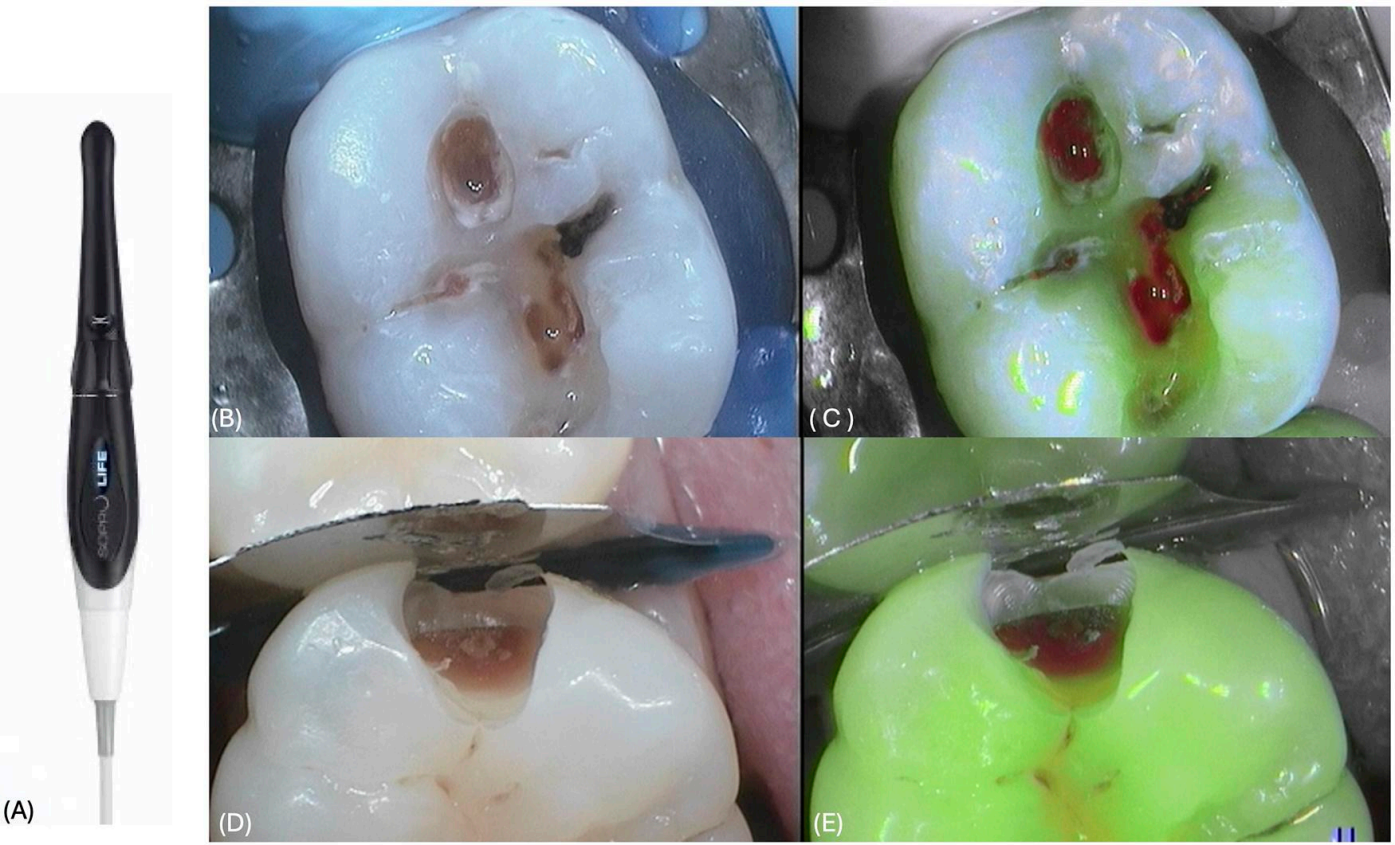
**(A)** Soprolife® (Acteon, France) fluorescence intra-oral camera; **(B)** Soprolife® daylight mode image during an occlusal caries lesion excavation; **(C)** Soprolife® fluorescence mode II image during an occlusal caries lesion excavation revealing the red fluorescent active carious tissue remaining; **(D)** Soprolife® daylight mode image during a proximal caries lesion excavation; **(E)** Soprolife® fluorescence mode II image during a proximal caries lesion excavation revealing the red fluorescent active carious tissue remaining.

Operating microscopes (Figures 1A, B) and intraoral cameras equipped with fluorescence capabilities (Figures 2–4) enable simultaneous evaluation of morphology and optical biomarkers. This integration represents an important step toward biologically informed diagnostics because clinicians can interpret structural findings within the context of tissue fluorescence and biochemical activity (9, 13).

### 4.3 Systems based on changes in light transmission, reflection or scaterring

#### 4.3.1 Light transmission technologies

Transillumination technologies exploit differences in light transmission caused by enamel demineralization. Sound enamel transmits light relatively efficiently because of its homogeneous mineral structure. Demineralization creates microporosities that increase scattering and reduce transmission. As a result, lesions appear as darkened areas against a brighter background. The principal advantages of transillumination include the absence of ionizing radiation, repeated use during monitoring, immediate image acquisition and a high patient acceptance. Several generations of transillumination systems have been developed (13).

Fiber-optic transillumination (FOTI), DIFOTI, and DIAGNOdent® (Kavo, Germany) were among the first radiation-free optical methods for caries detection. FOTI and DIFOTI rely on light transmission changes in demineralized tissue, whereas DIAGNOdent® uses laser-induced fluorescence. Despite their historical importance, these first-generation tools provide limited clinical information, cannot reliably assess lesion activity, may yield false positives, and have other diagnostic limitations. They have therefore been largely replaced by modern fluorescence imaging, near-infrared transillumination, and hybrid technologies with greater diagnostic accuracy and biological relevance (11, 13, 33).

#### 4.3.2 Near-infrared light transillumination (NILT)

Near-infrared light transillumination (NILT) represents the most important advancement within transillumination technologies. Systems such as DIAGNOcam®HD (Kavo, Germany) employ wavelengths around 780–850 nm. At these wavelengths, sound enamel exhibits significantly reduced scattering compared with visible light, allowing greater penetration and improved visualization of subsurface lesions (34). DIAGNOcam® HD is designed for approximal and occlusal lesion detection (Figure 2). Advantages include the improvement of proximal lesion detection, a greater penetration depth, a high image contrast, the absence of ionizing radiation and a real-time imaging.

NILT have demonstrated diagnostic performance comparable to bitewing radiography for proximal dentinal lesions. NILT is particularly useful for pediatric patients, pregnant patients, orthodontic monitoring, high-risk recall programs. However, NILT remains primarily a structural imaging modality. Although lesion extent can be visualized effectively, lesion activity cannot be directly assessed (13). Consequently, NILT is best considered complementary to fluorescence-based approaches that provide biological information.

### 4.4 Systems based on changes in fluorescence intensity or spectral peaks characteristics

Fluorescence technologies represent the most direct clinical translation of laboratory biophotonics. As discussed in Section 2, fluorescence signals arise from endogenous and exogenous fluorophores associated with: bacterial metabolism, porphyrin accumulation, collagen degradation, advanced glycation end-products and mineral changes. These biochemical origins distinguish fluorescence technologies from purely structural imaging systems (9, 13).

#### 4.4.1 Quantitative light-induced fluorescence (QLF)

Quantitative Light-Induced Fluorescence (QLF) is among the most extensively investigated fluorescence systems in cariology. The technology measures fluorescence loss associated with mineral depletion while simultaneously assessing red fluorescence associated with bacterial activity (Porphyrin signal). Modern QLF platforms such as QrayCam Pro® (Korea) and QrayPen® (Korea) generate quantitative parameters including fluorescence loss (ΔF), lesion area, red fluorescence intensity and mineralization changes (Figure 3).

A major advantage of QLF is its capacity for longitudinal monitoring. Applications include white spot lesion assessment, orthodontic demineralization, preventive studies, remineralization monitoring and clinical research. Because QLF provides objective numerical data, it is particularly valuable for monitoring disease dynamics over time (13).

#### 4.4.2 C50®, Soprolife® and SoproCare® (Acteon, France)

Among currently available fluorescence technologies, C50®, and previous versions Soprolife® and SoproCare® intraoral cameras, represent a translation of laboratory biophotonics into clinical dentistry. These systems integrate magnification, day-light imaging, fluorescence imaging, image capture and video recording within a single platform. C50® camera exhibits four imaging modes: Daylight, Daylight Boost, Caries and Perio mode. Using blue-light excitation around 450 nm, characteristic fluorescence patterns become visible (Figure 4):

- Acid-green fluorescence → sound dentin
- Bright red fluorescence → active dentinal caries
- Grey-green fluorescence with red shadows → tertiary or arrested dentin
- Dark green → Infected dentine to be removed.

An *in vivo* multicenter study of 628 occlusal fissures confirmed that the combination of magnification and fluorescence enhanced early caries detection, improved diagnostic confidence and provided detailed visual documentation that supported clinical decision-making and preventive management. The Soprolife® fluorescence camera demonstrated superior diagnostic performance compared with DIAGNOdent®, showing significantly higher overall accuracy (AUROC 0.81 in daylight mode and 0.79 in fluorescence mode vs. 0.67 for DIAGNOdent®) and excellent reproducibility (*κ* = 0.89–0.93) (35). These findings were further confirmed in a multicenter *in vivo* study involving children and adolescents, which demonstrated the strong diagnostic performance of the Soprolife® camera for the early detection of occlusal caries lesions (36).

**Figure 5:**
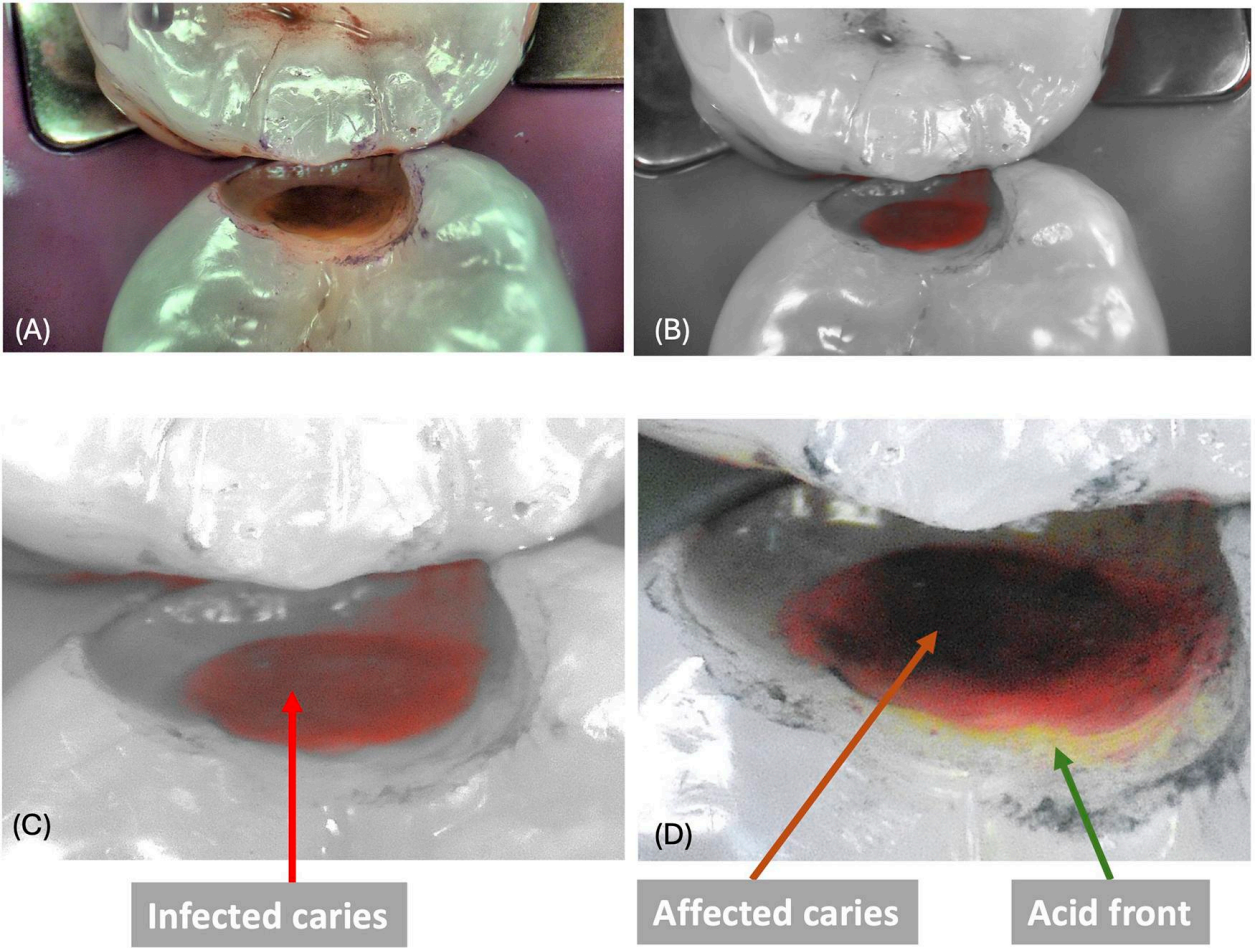
C50® (Acteon) intra-oral images. **(A)** Daylight image. **(B)** CarioMode: red fluorescent active caries. **(C)** CarioMode with higher magnification: red fluorescent active caries during excavation (red arrow). **(D)** CarioMode florescence image after selective caries removal: affected caries appears dark grey with red shadows red fluorescence (orange arrow) and the acid front (green arrow).

Importantly, these patterns correspond directly to molecular findings obtained from fluorescence spectroscopy, Raman analysis, and multiphoton investigations. This direct relationship between molecular biomarkers and clinical imaging makes these devices a particularly compelling example of translational biophotonics. The technology also forms the basis of the Light-Induced Fluorescence Evaluator for Diagnosis and Treatment (LIFEDT) (11, 35, 37, 38). Within this framework, fluorescence images are interpreted according to the activity, cavitation, cleanability and the biological status, rather than lesion depth alone This approach aligns closely with contemporary biological decision-making paradigms (8). Figure 5 illustrates the clinical steps that led to a selective caries removal guided by the dentine fluorescence, allowing tissue reservation, improving restorative adhesion potential and pulp vitality preservation.

#### 4.4.3 VistaCam® iX HDSmart (Dürr Dental, Germany)

**Figure 6:**
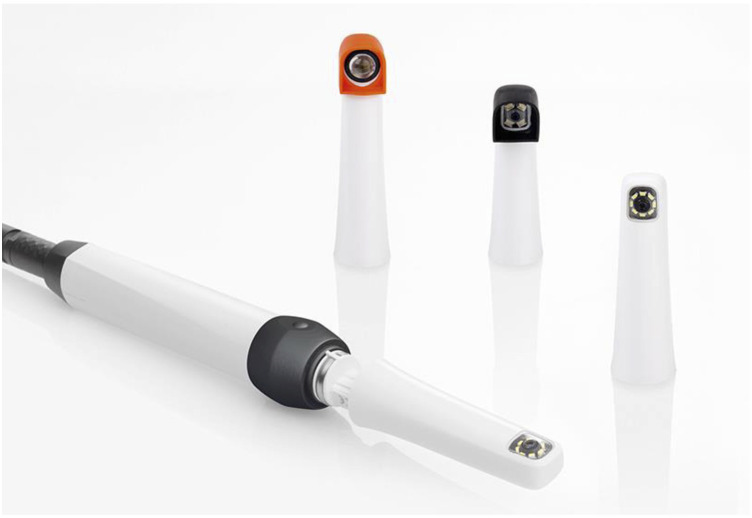
Vistacam® iX HD smart (Dürr Dental, Germany). [Source: www.duerrdental.com]

VistaCam® iX HD Smart combines, daylight HD view (1,280 × 1,024 resolution pixels), fluorescence imaging with quantitative analysis using violet-light excitation (405 nm) and Infrared proximal detection with three interchangeable heads (Figure 6).

VistaCam® iX HD Smart provides both digital image documentation and numerical lesion scoring (Table 2). This dual functionality improves interpretation and facilitates communication with patients. However, fluorescence measurements remain sensitive to surface hydration, plaque accumulation, surface staining and cleaning quality. Consequently, standardized acquisition protocols are essential for reproducible results (13).

**Table 2:** Cut off values of VistaCam® iX HD smart.

VistaCan score	Clinical interpretation
0–1	Healthy tooth enamel
1–1.5	Early stage of enamel caries
1.5–2	Deep enamel caries
2–2.5	Dentine caries
>2.5	Deep dentine caries

### 4.5 Future perspectives, emerging, hybrid and multimodal optical technologies

Although individual technologies provide valuable information, no single modality currently offers comprehensive assessment of lesion biology. This limitation can stimulate development of multimodal systems that combine complementary optical principles. Examples include:

- Fluorescence + OCT
- Raman spectroscopy + OCT
- Photothermal radiometry and luminescence (Canary System®)
- AI-assisted multimodal imaging platforms

Similarly, ongoing *in vitro* research efforts aim to combine Raman spectroscopy and OCT to achieve the ability to simultaneously assess molecular composition and lesion morphology of a biological tissue (39). Such approaches may ultimately provide the comprehensive biological information required for personalized diagnostics.

### 4.6 Current limitations of photonic caries detection technologies

Despite remarkable progress, several limitations remain. First, no single technology currently provides complete information regarding caries activity, cavitation, cleanability, lesion depth and biological status.

Second, diagnostic thresholds remain poorly standardized across systems and interpretation frequently depends on operator training and experience (13). Finally, advanced *in vitro* technologies such as OCT, Raman spectroscopy and multiphoton imaging remain associated with significant economic and technical barriers. Consequently, contemporary evidence supports the use of photonic technologies as adjuncts to comprehensive clinical examination rather than replacements for conventional diagnostics. Perhaps the most important evolution in dental biophotonics has been the shift from simple lesion detection toward biological interpretation. Contemporary technologies increasingly provide information regarding the bacterial activity, tissue integrity, collagen degradation, mineral status and lesion progression.

These capabilities support a more nuanced understanding of disease behavior and create the foundation for biological decision-making in modern cariology (8). The next section examines how photonic biomarkers contribute to lesion activity assessment, cavitation evaluation, cleansability analysis and risk-based clinical interpretation within the emerging framework of biologically guided diagnostics.

## 5 Photonic diagnostics and biological decision-making: a new paradigm in contemporary cariology

Historically, diagnostic systems were primarily designed to determine whether caries was present and whether operative intervention was required. Modern cariology, however, recognizes dental caries as a behavioral and a dynamic biological process whose progression depends on the balance between pathological and protective factors. Consequently, diagnosis must extend beyond **lesion detection** to include characterization of **lesion activity, cavitation status, accessibility to plaque control and progression risk** (8, 9). These parameters are critical because they directly influence clinical management decisions and monitoring strategies.

As explained in Section 2, unlike conventional radiography, which primarily reflects mineral density variations, photonic biomarkers may provide information regarding bacterial metabolism, collagen degradation, tissue organization and lesion dynamics (Table 3). Thus, the principal contribution of biophotonics to modern cariology may not be improved lesion detection alone, but rather the ability to support biological interpretation of disease.

**Table 3:** Translation of photonic properties into clinical caries detection technologies.

Photonic principle	Tissue property	Biomarker	Technology
Fluorescence	Endogenous fluorophores (sound tissues); porphyrins and pentosidine (carious tissues)	Green autofluorescence (sound tissues); red fluorescence (active carious tissues)	C50, Soprolife, VistaCam
SHG	Collagen organization	SHG intensity	Multiphoton microscopy
Raman spectroscopy	Molecular mineral organic composition, crystallinity, mineral ratio	Raman spectral peaks	Raman spectroscopy
NIR	Scattering	Contrast loss	DIAGNOcam HD

The Bioactive Dental Concept places particular emphasis on biological assessment as the foundation of a **clinical and biological decision-making framework** (9). Although frequently discussed within the context of bioactive restorative strategies, the conceptual framework itself is fundamentally diagnostic in nature. It proposes that clinicians should address four principal questions:

1. Is the lesion biologically active? Color change, texture
2. Is early cavitation present, visible and quantifiable?
3. Is the lesion accessible to plaque control or professional cleaning Plaque accumulation
4. What is the patient's susceptibility to future disease? Patient compliance

These questions represent a shift from a lesion-centered model toward a patient-centered and biology-centered approach (5, 9, 40). Importantly, no single conventional diagnostic modality can answer all four questions reliably (Table 4). Radiographs provide information regarding lesion depth but not activity. Visual examination provides information regarding surface characteristics but limited insight into subsurface biological processes. Photonic technologies contribute additional layers of information that can support interpretation of these clinically relevant variables.

**Table 4:** Photonic devices and their clinical efficiency.

Technology	Activity	Cavitation	Cleansability	Monitoring	Image Capture
DIAGNOdent/cam	++ false positive	−	−	+	−/+
QLF	+++	+	+	+++	+++
Soprolife/ C50	+++	+++	++	+++	+++
VistaCam	++	++	+	++	+++
NILT	−	+	+	++	+++

(−) not available/not applicable; (+) limited capability; (++) moderate capability; (+++) high capability; (++++) very high capability. The ratings are intended to provide a comparative overview and do not represent quantitative performance metrics or validated diagnostic accuracy scores.

Although clinically valuable, these observations remain subjective and may demonstrate considerable inter-examiner variability (13).

Fluorescence technologies offer a potentially valuable adjunctive approach because their signals originate from biochemical processes associated with active disease (**Fluorescence-based activity assessment**). Laboratory research has demonstrated associations between red fluorescence and porphyrin accumulation, bacterial metabolism, collagen degradation, pentosidine formation and advanced glycation end-products (18). Because these molecules are frequently associated with metabolically active lesions, fluorescence imaging may provide indirect information regarding lesion activity that is not accessible through radiography. Systems such as C50®, Soprolife®, QLF and VistaCam® therefore occupy a unique position within contemporary diagnostics by providing biologically relevant optical information rather than purely structural data.

**Figure 7:**
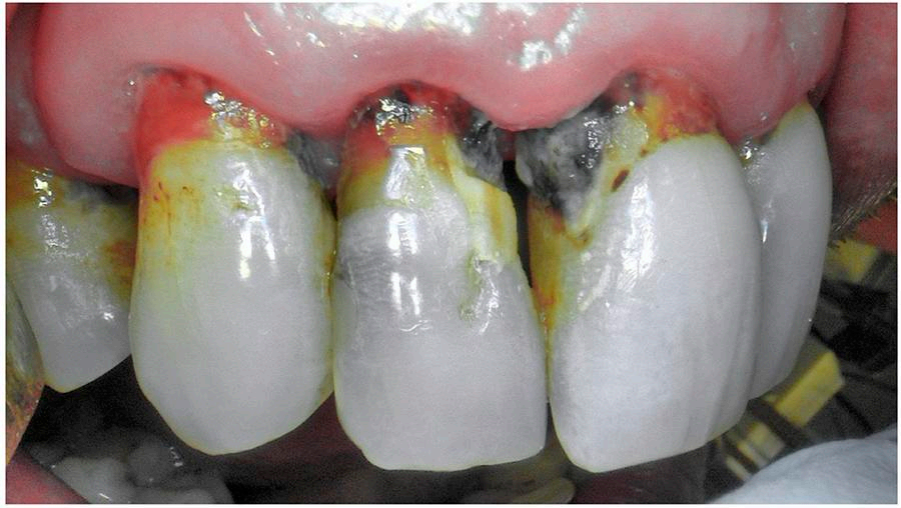
C50® Perio mode intraoral image illustrating the surface texture alteration.

The distinction between cavitated and non-cavitated lesions remains fundamental to contemporary caries classification systems (4, 5, 8, 9). **Cavitation** significantly alters biofilm retention, plaque accessibility, cleanability and lesion progression dynamics. Visual examination remains the primary method for cavitation assessment. However, optical technologies and magnification can substantially improve detection of subtle surface breakdown. Magnification systems enhance visualization of surface discontinuities, fissure morphology, micro-cavitations and surface texture alterations (Figure 7).

Similarly, fluorescence cameras provide simultaneous information regarding surface morphology, lesion extent and biological activity. This combination may improve diagnostic confidence when cavitation status is uncertain.

**Figure 8:**
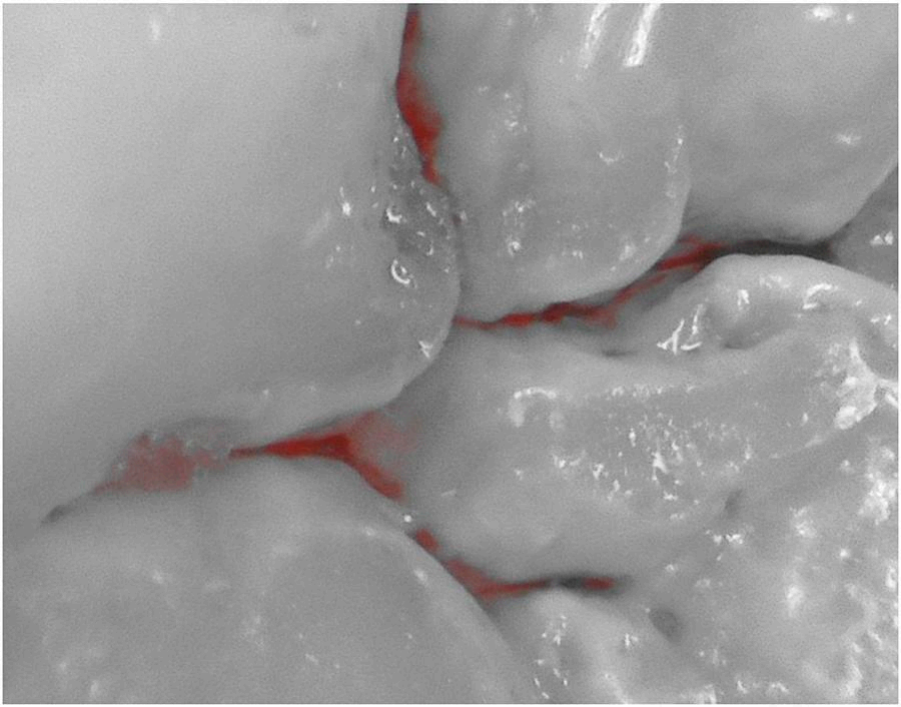
C50® fluorescence image of an occlusal fissure with residual active biofilm (red signal) after cleaning using an airflow with erythritol powder.

Historically, diagnostic technologies focused primarily on lesion detection and severity. However, lesion accessibility strongly influences biological behavior. Lesions located within areas readily accessible to oral hygiene measures may remain stable or arrest despite evidence of demineralization. Conversely, lesions situated within plaque-retentive environments frequently remain active and **cleanability and accessibility** can be considered as emerging diagnostic variable (Figure 8) (5, 8–10).

**Figure 9:**
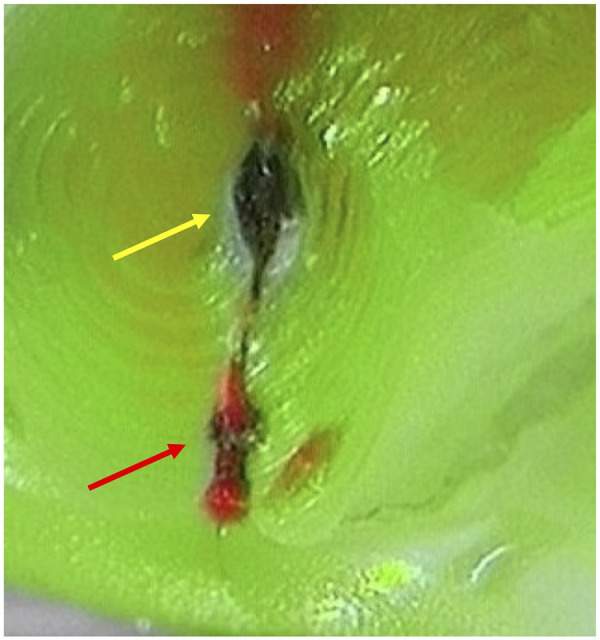
Soprolife® fluorescence image illustrating the complexity of the cleanability evaluation: active carious fissure (red arrow -ref fluorescence) and non-active-carious fissure (yellow arrow).

Photonic technologies can contribute to cleanability assessment by improving visualization of plaque stagnation zones, surface anatomy, fissure morphology, early cavitation and biofilm-associated fluorescence (Figures 8, 9). In this context, optical imaging contributes not only to lesion characterization but also to ecological assessment of the lesion environment.

Modern cariology increasingly recognizes that lesions cannot be interpreted independently from the patient in whom they occur. Individual caries risk substantially influences lesion behavior and progression (salivary dysfunction, dietary habits, biofilm composition, fluoride exposure, medical conditions, behavioral factors etc.) (8).

For example:

- Persistent fluorescence activity may indicate ongoing disease despite preventive measures.
- Stable fluorescence patterns may suggest lesion arrest.
- Serial NILT examinations may reveal structural progression.
- OCT monitoring may identify morphological changes before cavitation develops.

Thus, photonic biomarkers complement conventional **risk assessment** and contribute to **individualized disease characterization**.

A major limitation of traditional diagnostics is that they frequently provide only isolated observations at specific time points. Photonic technologies offer a significant advantage because they facilitate longitudinal monitoring. Systems such as QLF, ® C50®, Soprolife® and VistaCam®HD (cut off values) allow repeated image acquisition and comparison over time. This capability enables assessment of lesion progression and stabilization, changes in fluorescence intensity, alterations in mineralization and morphological evolution. Such **monitoring** aligns closely with the objectives of minimally invasive and biologically driven cariology.

## 6 Discussion

Over the past decades, dental biophotonics has moved from laboratory research to a clinically useful field that increasingly supports everyday dental practice. Techniques such as fluorescence spectroscopy, Raman spectroscopy, SHG, 2PEF and OCT have shown that dental tissues contain intrinsic optical signals related to mineralization, collagen structure, bacterial activity and lesion progression.

**Figure 10:**
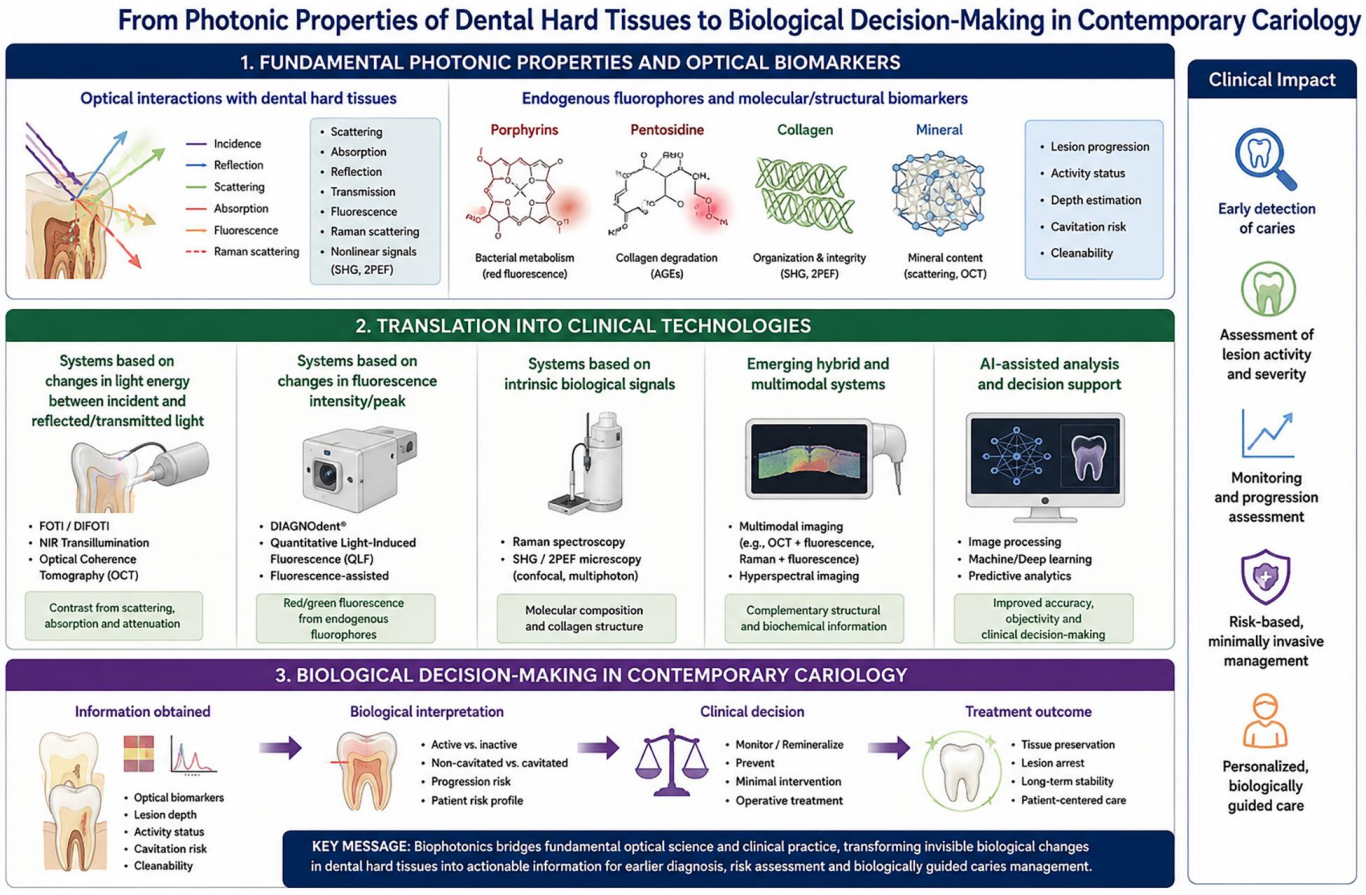
Graphical summary: from photonic properties of dental hard tissues to biological decision-making in contemporary cariology.

A major achievement of the field is the translation of these findings into clinical tools. Systems such as REVEAL Loupes, DIAGNOcam®, QLF, Soprolife®, C50, and VistaCam® give clinicians access to information that previously required laboratory or histological analysis (13). Unlike conventional radiography, which mainly shows mineral loss, biophotonic methods also provide structural, biochemical and functional information. This is especially valuable in modern cariology, where lesion activity and biological behavior matter as much as lesion depth. This shift from signal detection to biological interpretation marks an important change in dental diagnostics (Figure 10).

A key debate in modern cariology is whether diagnosis should focus mainly on structure or also on biology. The traditional structural model relies on lesion shape, cavitation, and radiographic appearance, with decisions based mostly on lesion depth and anatomical extent. Conventional radiography, visual-tactile examination and transillumination mainly follow this approach (12). In contrast, the biological model focuses on lesion activity, biofilm ecology, mineral changes, and the risk of progression (8, 9). Lesions with similar depth may behave very differently biologically, so structure alone is not enough for modern caries management. Photonic technologies help bridge these two views: NILT and OCT mainly provide structural information, while fluorescence-based methods reveal biological signals linked to bacterial metabolism and tissue breakdown. Raman spectroscopy and multiphoton microscopy go further by connecting optical signals to molecular processes. Rather than replacing structural diagnosis, biological assessment should complement it and future cariology will likely combine both approaches. Despite major progress, several challenges still limit wider clinical use. One major barrier is the lack of universally accepted thresholds for interpreting fluorescence, Raman signals, SHG/2PEF ratios and other optical biomarkers. Although many studies show correlations with lesion features, standardized clinical criteria are still missing. This makes comparison between studies difficult and slows clinical adoption. Assessing lesion activity is essential but still poorly standardized. Red fluorescence is a promising marker because it is associated with porphyrins, pentosidine and bacterial metabolism. However, its intensity can also be affected by plaque, contamination, hydration and staining (13, 18). More longitudinal studies are needed before this type of assessment can be standardized.

Advanced methods such as Raman spectroscopy, multiphoton microscopy and SHG imaging provide rich biological information, but their clinical use is still limited by cost, complexity and long acquisition times. Smaller and simpler systems will be needed for broader adoption. Many published studies are still cross-sectional or laboratory based. Future research should prioritize prospective longitudinal studies on lesion progression, arrest, predictive value, patient-centered outcomes and clinical decision-making. The future of dental biophotonics will likely depend on combining multiple technologies rather than relying on a single diagnostic method.

Recent studies show that deep-learning algorithms can detect carious lesions with performance close to expert clinicians (41–43). In the future, AI may help with lesion classification, activity assessment, risk prediction and follow-up. However, important limitations remain. Most studies are retrospective and performed under controlled conditions. Training databases are often small, heterogeneous, or not fully representative of real clinical practice. AI performance can also be affected by image quality, anatomical variation, restorations and artifacts. For now, these factors limit the clinical generalizability of many AI tools. No single optical method can currently assess lesion biology in a complete way. Future multimodal platforms may combine fluorescence imaging, NILT, OCT, Raman spectroscopy, SHG biomarkers and/or AI to evaluate structure, composition, and biological activity at the same time.

Personalized dentistry is also expected to shape oral healthcare. Future diagnostic systems may combine optical biomarkers with salivary analysis, microbiome data, risk assessment and patient behavior to build individualized disease profiles and guide personalized care.

## 7 Conclusion

The intrinsic photonic properties of enamel and dentin provide clinically relevant optical biomarkers that reflect mineralization, collagen organization and bacterial activity during the caries process. Advances in fluorescence imaging, Raman spectroscopy, multiphoton microscopy, near-infrared transillumination, and optical coherence tomography have enabled the successful translation of these fundamental optical phenomena into clinically applicable diagnostic technologies.

Although no single modality provides a comprehensive assessment of lesion biology, complementary optical approaches improve early detection, lesion characterization, and longitudinal monitoring, thereby supporting minimally invasive and biologically guided caries management. Future integration of multimodal imaging, artificial intelligence, and quantitative optical biomarkers is expected to further enhance the precision and clinical utility of photonic diagnostics.

Overall, biophotonics is reshaping contemporary cariology by bridging fundamental optical science with clinical decision-making and enabling earlier, more accurate, and biologically informed diagnosis of dental caries.
